# Evaluation Challenges in the Validation of B7-H3 as Oral Tongue Cancer Prognosticator

**DOI:** 10.1007/s12105-020-01222-3

**Published:** 2020-09-21

**Authors:** Meri Sieviläinen, Anna Maria Wirsing, Aini Hyytiäinen, Rabeia Almahmoudi, Priscila Rodrigues, Inger-Heidi Bjerkli, Pirjo Åström, Sanna Toppila-Salmi, Timo Paavonen, Ricardo D. Coletta, Elin Hadler-Olsen, Tuula Salo, Ahmed Al-Samadi

**Affiliations:** 1 Department of Oral and Maxillofacial Diseases, Clinicum, University of Helsinki, Helsinki, Finland; 2 Translational Immunology Program, Faculty of Medicine, University of Helsinki, Helsinki, Finland; 3 Department of Medical Biology, Faculty of Health Sciences, UiT The Arctic University of Norway, Tromsø, Norway; 4 Cancer Research and Translational Medicine Research Unit, University of Oulu, Oulu, Finland; 5 Medical Research Center Oulu, Oulu University Hospital, University of Oulu, Oulu, Finland; 6 Department of Otorhinolaryngology, University Hospital of North Norway, Tromsø, Norway; 7 Skin and Allergy Hospital, Helsinki University Hospital and University of Helsinki, Helsinki, Finland; 8 Department of Pathology, Faculty of Medicine and Health Technology and Fimlab laboratories, Tampere University and Tampere University Hospital, Tampere, Finland; 9 Department of Oral Diagnosis, Piracicaba Dental School, University of Campinas, Piracicaba, São Paulo, Brazil; 10 The Public Dental Health Competence Center of Northern Norway, Tromsø, Norway; 11 University of Helsinki Central hospital, Helsinki, Finland

**Keywords:** Oral tongue squamous cell carcinoma, B7-H3, Tumor infiltrating lymphocytes, Immune checkpoint, Replication crisis.

## Abstract

B7-H3 was the only molecule identified with prognostic potential from a recent systematic review of the prognostic value of immune checkpoints in oral cancer. We aimed to validate this finding in a multicenter international cohort. We retrospectively retrieved 323 oral tongue squamous cell carcinoma (OTSCC) samples from three different countries (Brazil, Finland, and Norway) for immunostaining and scoring for B7-H3. We evaluated tumor immunogenicity by analyzing the amount of tumor-infiltrating lymphocytes and divided the tumors into immune hot and cold. To increase the reliability of the results, both digital and manual visual scoring were used. Survival curves were constructed based on the Kaplan-Meier method, and the Cox proportional hazard model was utilized for univariate and multivariate survival analysis. B7-H3 expression was not significantly associated with overall or disease-specific survival in the whole OTSCC cohort. When divided into immune hot and cold tumors, high B7-H3 expression was significantly associated with poor disease-specific and overall survival in the immune hot group, depending on the scoring method and the country of the cohort. This was achieved only in the univariate analysis. In conclusion, B7-H3 was a negative prognosticator for OTSCC patient survival in the subgroup of immune hot tumors, and was not validated as a prognosticator in the full cohort. Our findings suggest that the immune activity of the tumor should be considered when testing immune checkpoints as biomarkers.

**Electronic supplementary material:**

The online version of this article (doi:10.1007/s12105-020-01222-3) contains supplementary material, which is available to authorized users.

## Introduction

The incidence of oral together with lip squamous cell carcinoma (OSCC) is unfortunately increasing. In 2018, the number of new cases worldwide was approximately 350,000 with an annual mortality of approximately 180,000 [1]. This increase is not associated with an increase in the 5-year survival rate, which remains at approximately 50% for most countries [1, 2]. Therefore, there is a need for new treatment and therapeutic approaches. OSCC arising from the tongue (OTSCC) is the most aggressive subgroup of oral cancers and is characterized by high rates of metastasis and mortality [3]. OTSCC carcinogenesis is traditionally associated with heavy alcohol and tobacco use [4]. In addition, evasion of the host immune response to the tumor has been recognized as a key feature in the carcinogenesis process, and tumors with low infiltration of immune cells respond more poorly to immune-based cancer therapies [5]. These findings have led the way to a novel classification of tumors into two categories, “hot” (or inflamed) and “cold” (or non-inflamed), based on quantification of tumor-infiltrating lymphocytes (TILs) [6]. Head and neck cancers are generally highly infiltrated by lymphocytes and are thus immune hot; however, the poor patient survival suggests that the anti-tumor immune response is ineffective [7].

Immune checkpoints play a predominant role in the initiation of CD4^+^ and CD8^+^ T cell-dependent immune responses by regulating interactions between co-stimulatory ligands and their receptors [8]. Ligand members of the B7/CD28 superfamily, such as B7-H1 (PD-L1) and B7-H3, can modulate the initiation by either amplifying or inhibiting co-stimulatory signals. PD-L1 overexpression inhibits the activation of functional T-cells [9] and PD-1/PD-L1 axis inhibition has been adopted as a therapeutic approach for OSCC [10]. On the other hand, B7-H3 has no identified receptors and is theorized to be involved in both co-stimulation and co-inhibition of T cells [11]. In vitro, B7-H3 increases activity of CD8 + T cells but also inhibits T-cell proliferation and reduces secretion of relevant immune mediators such as interferon-γ, tumor necrosis factor α, and other cytokines [12, 13].

Several studies have been conducted to determine the prognostic value of immune checkpoints in oral cancer [14]. In our recent systematic review, B7-H3 showed evidence as an adverse prognostic factor in OSCC, while other immune checkpoints were either studied once or had controversial results [14]. According to Almangush and co-authors, hundreds of biomarkers have been studied as prognostic markers for OSCC, but none are in clinical use [15]. This may be due to several factors, mainly missing validation, as among the 12 immune-modulating molecules investigated thus far, only four had been studied more than once.

Since B7-H3 was the only immune checkpoint molecule that showed a potential role in the prognostication of OSCC, we sought to validate this result in a multicenter international cohort study.

## Methods and Materials

This study was performed according to the REMARK guidelines for tumor marker prognostic studies [16].

### Patient Samples

This study examined a total of 323 retrospective OTSCC samples obtained during tumor surgery from three different countries: 147 formalin-fixed paraffin-embedded (FFPE) whole-section samples from Finland (Oulu and Tampere University hospitals, collected during 1990–2010), 132 FFPE tumor microarray (TMA) samples from Norway (the University hospitals of Oslo, Bergen, Trondheim and Tromsø, collected during 2005–2009), and 44 FFPE whole-section samples from Brazil (UOPECCAN and CEONC Cancer Hospitals in Cascavel-Parana, collected during 2008–2014). Patients received treatment according to the respective national guidelines. In Finland, the data inquiry was approved by the National Supervisory Authority for Welfare and Health (VALVIRA) and the Ethics Committee of the Northern Ostrobothnia Hospital District (statement #8/2006, amendment 19/10/2006). The OTSCC sample collection in Brazil was approved by the Human Research Ethics Committee of the Piracicaba Dental School, University of Campinas. In Norway, the study was approved by the Institutional Review Board of Northern Norwegian Regional Committee for Medical Research Ethics (REK Nord) with validated approval for all hospitals (Protocol number REK Nord; 2013/1786 and 2015/1381). The clinical and demographic parameters of the patients are presented in Table 1.

**Table 1:** Demographic and clinicopathological parameters of the oral tongue squamous cell carcinoma patients

Patient clinical data	No. of patients (%) n = 323	Brazil n = 44	Finland n = 147	Norway n = 132
Age				
< 60	135 (40.8)	29 (65.9)	63 (42.9)	43 (32.6)
≥ 60	188 (56.8)	15 (34.1)	84 (57.1)	89 (67.4)
Range	17–99	31–83	17–99	25–90
Mean	62.60	48.4	63.1	64.3
Median	63	49	65	65
Sex				
Male	192 (58.0)	37 (84.1)	76 (51.7)	79 (59.8)
Female	131 (39.6)	7 (15.9)	71 (48.3)	53 (40.2)
Tumor grade				
I-II, Mild to moderate	265 (82.0)	38 (86.4)	114 (77.6)	113 (85.6)
III, Poor	52 (16.1)	6 (13.6)	33 (22.4)	13 (9.8)
Missing*	6 (1.9)	0 (0)	0 (0)	6 (4.5)
Tumor stage				
T1-T2	233 (72.1)	37 (84.1)	89 (60.5)	102 (77.3)
T3-T4	79 (24.5)	7 (15.9)	58 (39.5)	10 (7.6)
Missing*	11 (3.4)	0 (0)	0 (0)	11 (8.3)
Neck metastasis				
N1	108 (33.4)	19 (43.2)	74 (50.3)	28 (21.2)
N0	204 (63.2)	25 (56.8)	72 (49.0)	93 (70.5)
Missing*	11 (3.4)	0 (0)	0 (0)	11 (8.3)
Treatment				
Surgery	116 (35.9)	13 (29.5)	73 (49.7)	30 (22.7)
Surgery and radiotherapy	131 (40.6)	15 (34.1)	22 (15.0)	94 (71.2)
Surgery, radio- and chemotherapy	76 (23.5)	16 (36.4)	52 (35.3)	8 (6.1)
Recurrence				
No recurrence	224 (69.3)	33 (75.0)	88 (60.0)	103 (78.0)
Recurrence	91 (28.2)	11 (25.0)	58 (39.5)	21 (15.9)
Missing*	8 (2.5)	0 (0)	0 (0)	8 (2.5)

*Norwegian cases have 11 samples with some missing clinical data

### Immunohistochemical Staining

For optimizing the staining protocol, we used the following two antibodies for B7-H3: rabbit anti-human B7-H3 (D9M2L, 1:200, Cell Signaling technology, Leiden, Netherlands) and goat anti-human B7-H3 (AF1027, 1:1000, R&D-systems, Minneapolis, MN, USA). Antibody selection was based on two published articles [8, 17]. Three researchers (M.S. junior trainee; A.A-S., senior trainee; and T.S., oral pathologist) evaluated the staining with an optical microscope (Leica DM6000 together with Leica DFC365-FX camera, Leica Biosystems, Newcastle, UK). Both antibodies had the same staining pattern with slight differences in staining intensity (Online Resource 1); therefore both were used in this study. The Finnish samples were stained with rabbit anti-human antibody and the Norwegian and Brazilian samples were stained with goat anti-human antibody.

For the rabbit antibody, Dako Real EnVision Detection system K5007 kit (Dako, Carpinteria, CA) was used for staining. After deparaffinization, epitopes were retrieved in Tris-EDTA buffer (pH 9) for 15 minutes using a microwave and followed by cooling at room temperature for 20 minutes. Dako Peroxidase blocking solution S2023 was next applied for 15 minutes. Sections were then incubated with the rabbit B7-H3 primary antibody for 1 hour at room temperature followed by Dako HRP for 30 minutes at room temperature.

For the goat-based antibody, we used a goat on rodent HRP-polymer detection kit (Biocare Medical, Pacheco, CA). After deparaffinization, antigens were retrieved in citrate buffer (Dako) for 15 minutes using a microwave and followed by cooling at room temperature for 20 minutes. Dako peroxidase blocking solution S2023 was then applied for 15 minutes. Sections were then incubated with the goat B7-H3 primary antibody for 30 minutes. Goat probe from the detection kit was added for 15 minutes and followed by goat on rodent HRP polymer for 15 minutes.

Both rabbit and goat sections were then incubated with chromogen DAB for color formation for 15 minutes and washed in dH_2_O for 5 minutes. The slides were then counterstained with Mayer’s hematoxylin solution (Sigma-Aldrich, St. Louis, MO, USA) and mounted in Mountex (HistoLab, Gothenburg, Sweden).

Slides were scanned using a Leica Aperio AT2 (Leica Biosystems) to be analyzed using QuPath software.^18^ The specificity of each staining was confirmed with staining controls.

### Assessment of B7-H3 Expression using Manual Visual Scoring

Two researchers for each cohort (Finland: A.H., junior trainee, M.S. junior trainee; Norway: A.W., senior trainee, E. H-O., senior trainee; Brazil: P.C., senior trainee, P.Å., senior trainee) evaluated all scanned samples independently and then jointly for consensus while blinded to any clinical data.

**Fig. 1:**
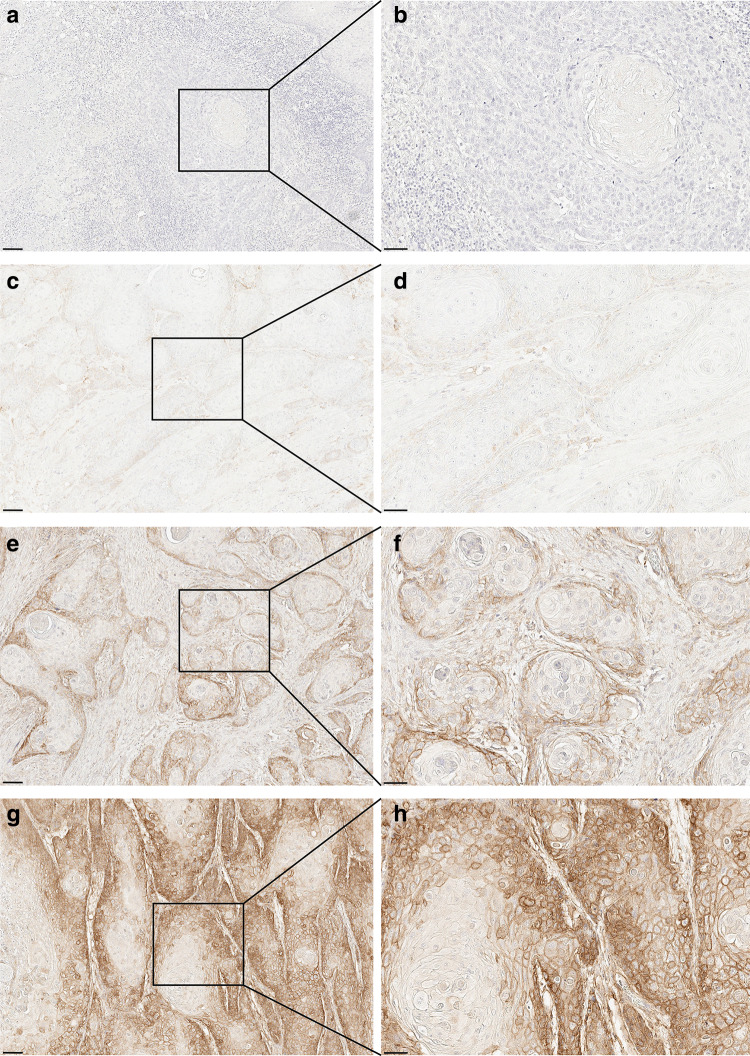
B7-H3 expression in oral tongue squamous cell carcinoma cells: **a**-**b**. Negative staining (intensity = 0), **c**-**d**. Mild staining (intensity = 1), **e**-**f**. Moderate staining (intensity = 2), **g**-**h**. Strong staining (intensity = 3). Scale bar **a**, **c**, **e**, **g**, 100 µm; **b**, **d**, **f**, **h**, 50 µm

Staining intensity was evaluated as 0–3 (0: negative, 1: weak, 2: moderate, and 3: strong; Fig. 1) and the staining area was evaluated as 0–3 (0: 0%, 1: 0 > 25%, 2: 25 > 50%, 3: >50%). The staining index was calculated as a sum of the two scores. The Norwegian TMA samples did not allow a meaningful evaluation of the staining area, thus only staining intensity is reported from these samples.

### Assessment of B7-H3 Expression using Digital Scoring

In addition to the traditional manual visual scoring, we sought to validate our results by using a free, automated analysis software, QuPath [18]. Two researchers (M.S. and P.C.) with coding experience developed the automated scoring protocol. First, the program was calibrated to detect colors by estimating the staining vectors. All FFPE and TMA samples had similar modal RGB and DAB values. Second, the classifier was taught to recognize cancer and stromal cells by choosing five areas of tumor and five areas of stroma for ten slides. Cell and membrane detection was performed according to the developer instructions (Online Resource 2). Third, the classifier was calibrated by comparing different mean DAB OD values for different slides from different countries to determine the thresholds to be used. Both researchers performed the analysis first independently and then agreed on the values to be used on all slides (Online Resource 2). The classifier was then saved and the script was coded. One researcher (Finland: M.S.; Brazil: P.C.) for each cohort selected 5 representative areas of the invasive front in FFPE samples, TMA was taken as a whole, and ran the automated software. The scripts are available in Online Resource 3 and 4. Results were in the form of H-score and were extracted for survival analysis.

### Tumor-Infiltrating Lymphocytes (TILs) Scoring

Two researchers (A.H.; junior trainee, M.S.; junior trainee) evaluated the presence of TILs in the Brazilian and Finnish samples independently and divided the cases into immune hot and cold while remaining blinded to any clinical data [6]. Disagreements between evaluators were resolved by an experienced researcher (A.A-S; senior trainee). Scoring was conducted as previously described [19]. Based on this study, only stromal TILs were assessed. The scoring was defined as the percentage of stroma occupied by lymphocytes (0%, 5%, 10%, 20%, 30%, 40%, and ≥ 50%). Only areas directly related to the invasive front were included in the estimation. Areas of fibrosis, central necrosis, or artefacts were excluded. Norwegian cases were not scored for the TILs as they were TMAs, which do not allow the full evaluation of the tumor stroma.

### Inter-Rater Reliability

A κ coefficient was calculated to measure the agreement between evaluators. Interpretation of the κ coefficient was based on Landis and Koch 1977 (poor agreement: less than 0.20, fair agreement: 0.20–0.40, moderate agreement: 0.40–0.60, substantial agreement: 0.60–0.79, and almost perfect agreement: 0.80–1.00) [20]. The highest agreement in the manual visual scoring of B7-H3 between the evaluators was in the Norwegian (κ-value 0.84, 95% confidence interval 0.81–0.89) and Brazilian (κ -value 0.83, 95% confidence interval 0.70–0.97) cases, reaching almost perfect agreement followed by Finnish cases (κ -value 0.57, 95% confidence interval 0.51–0.64) with moderate agreement. For TIL scoring, there was moderate agreement between the evaluators (κ-value 0.55, 95% confidence interval 0.51–0.59).

### Statistical Analysis

After scoring, cases were divided into high and low expression using the median as the cut-off point. We also performed the analysis by calculating the optimal cut-off point [21], but this did not change the results (data not shown). Additionally, the cases were divided into immune hot if TILs were ≥ 20%, and cold if median TILs were < 20%, based on a previous study [19]. The κ coefficient was calculated and the prognosis of patients in relation to overall survival and disease-specific mortality was analyzed using SPSS software program version 21.0 (IBM SPSS Statistics, SPSS INC, Chicago, IL, USA). Life tables were calculated according to the Kaplan-Meier method. Survival curves were compared with the log-rank test. Univariate and multivariate survival analyses were performed with Cox’s proportional hazards model. In multivariate analysis, the results were adjusted for age, sex, grade, stage, and lymph node metastasis. Statistical significance was set at p < 0.05.

## Results

### B7-H3 Expression in OTSCC Samples

**Fig. 2:**
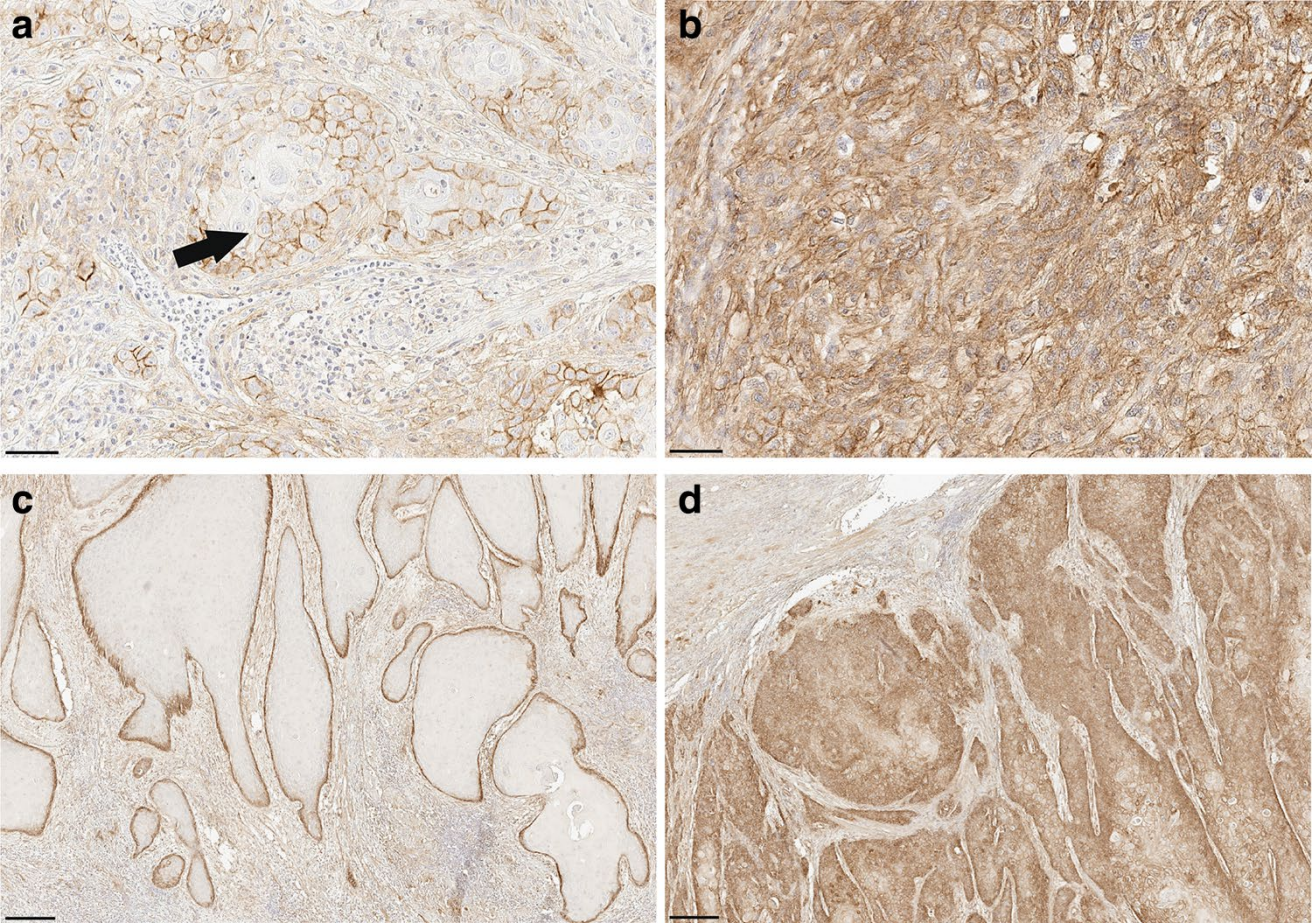
B7-H3 location and expression pattern. B7-H3 is mainly located at the cancer cell membrane (**a**) and sometimes was observed in the cancer cell cytoplasm (**b**). In most of the slides, B7-H3 was expressed only at the periphery of the tumor island (**c**) and in some cases was expressed in the whole tumor island (**d**). Scale bar **a**, **b** 50 μm; **b**, **d** 250 μm

B7-H3 was mainly expressed at the membrane of the cancer cells (Fig. 2a). Staining was also seen in the cytoplasm in some heavily stained samples, (Fig. 2b). The staining was mainly concentrated at the periphery of the tumor islands (Fig. 2c). However, the whole tumor island was positive in some cases (Fig. 2d).

### B7-H3 Expression is not Associated with Survival of OTSCC Patients

During follow-up, 120 patients died of OTSCC, 56 patients died of other causes, and 147 patients were alive at the end of the follow-up period. Median follow-up time was 40 months (range: 0–252 months). B7-H3 expression was not significantly associated with OTSCC mortality (Table 2). We performed the analysis for each country separately to determine if differences in population or laboratories had any impact on the results. All subgroups showed similar results to the combined data, which indicated that B7-H3 is not significantly associated with disease-specific or overall survival (Table 2). Even with the optimal cut-off points, no significance was found in any of the subgroups (data not shown).

**Table 2:** Univariate survival analysis for disease specific and overall survival based on B7-H3 expression. Low B7-H3 is taken as a reference

	All samples (n = 323)			
	Disease specific survival		Overall survival	
	HR (95%CI)	p-value	HR (95%CI)	p-value
Digital scoring	0.91 (0.63–1.30)	0.58	1.00 (0.75–1.35)	0.98
Manual visual scoring intensity	0.97 (0.67–1.40)	0.88	1.07 (0.79–1.45)	0.64

	Brazil (n = 44)			
	Disease specific survival		Overall survival	
	HR (95%CI)	p-value	HR (95%CI)	p-value
Digital scoring	1.29 (0.56–2.94)	0.55	1.91 (0,89-4.11)	0.10
Manual visual scoring index	1.02 (0.45–2.33)	0.97	1.17 (0.56–2.48)	0.68
Manual visual scoring intensity	0.97 (0.43–2.21)	0.94	1.24 (0.59–2.62)	0.57
Manual visual scoring area	1.13 (0.49–2.57)	0.77	1.21 (0.57–2.56)	0.61

	Finland (n = 147)			
	Disease specific survival		Overall survival	
	HR (95%CI)	p-value	HR (95%CI)	p-value
Digital scoring	1.19 (0.67–2.12)	0.55	1.05 (0.67–1.65)	0.82
Manual visual scoring index	1.4 (0.78–2.5)	0.25	1.32 (0.84–2.09)	0.22
Manual visual scoring intensity	0.75 (0.42–1.34)	0.33	0.74 (0.47–1.17)	0.20
Manual visual scoring area	1.72 (0.97–3.39)	0.10	1.54 (0.91–2.58)	0.10

	Norway (n = 132)			
	Disease specific survival		Overall survival	
	HR (95%CI)	p-value	HR (95%CI)	p-value
Digital scoring	1.16 (0.67–2.03)	0.60	1.26 (0.79–2.01)	0.33
Manual visual scoring intensity	0.91 (0.52–1.59)	0.75	1.01 (0.63–1.6)	0.97

### High B7-H3 Expression is Associated with Poor Survival in the Immune Hot Subgroup of OTSCC Patients

**Fig. 3:**
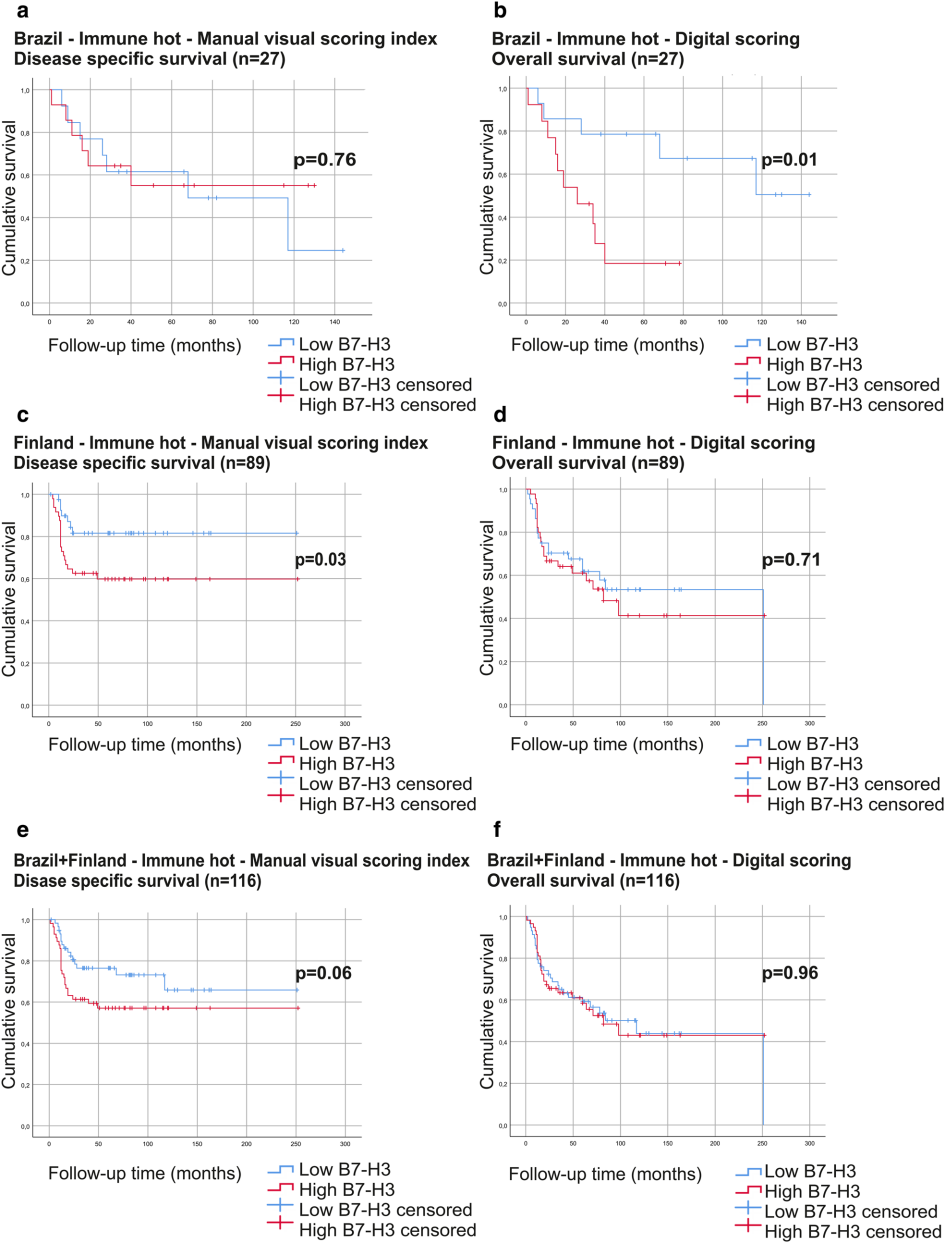
Survival curves of the B7-H3 high and low expression for immune hot cases. Kaplan-Meier curves representing the Finnish cases for disease-specific survival (**a**) and overall survival (**b**), Brazilian cases for disease-specific survival (**c**) and overall survival (**d**) and all cases (Finnish and Brazilian) for disease-specific survival (**e**) and overall survival (**f**)

As B7-H3 mainly exerts its effects on lymphocytes, we divided the cancer samples into immune hot or cold (high or low amount of TILs, respectively) and performed the analysis for each group separately. The TMA samples from Norway could not be separated into these two groups. In immune hot cases, high B7-H3 expression associated with low overall survival in digitally scored Brazilian samples, and with low disease-specific survival in manually visually scored (scoring index and area) Finnish cases (Table 3; Fig. 3). The significant association was not observed in multivariate analysis (data not shown). In immune cold cases, no significant correlation was found between B7-H3 expression and patient survival in any of these analyses (Table 3).

**Table 3:** Univariate survival analysis results for B7-H3 expression in immune hot and cold samples. Low B7-H3 is taken as a reference

Brazil + Finland (n = 191)	Immune hot cases (n = 116)				Immune cold cases (n = 75)			
	Disease specific survival		Overall survival		Disease specific survival		Overall survival	
	HR (95%CI)	p-value	HR (95%CI)	p-value	HR (95%CI)	p-value	HR (95%CI)	p-value
Digital Scoring	1.05 (0.56–1.97)	0.87	1.01 (0.59–1.72)	0.96	0.77 (0.38–1.57)	0.47	0.72 (0.41–1.26)	0.25
Manual Visual Scoring Index	1.85 (0.97–3.53)	0.06	1.47 (0.87–2.49)	0.15	0.66 (0.32–1.35)	0.25	0.89 (0.51–1.57)	0.69
Manual Visual Scoring Intensity	0.71 (0.38–1.33)	0.29	0.71 (0.41–1.19)	0.19	0.77 (0.37–1.58)	0.47	0.89 (0.49–1.61)	0.71
Manual Visual Scoring Area	1.78 (0.90–3.51)	0.09	1.42 (0.82–2.46)	0.21	0.74 (0.32–1.58)	0.44	0.92 (0.47–1.79)	0.81

Brazil (n = 44)	Immune hot cases (n = 27)				Immune cold cases (n = 17)			
	Disease specific survival		Overall survival		Disease specific survival		Overall survival	
	HR (95%CI)	p-value	HR (95%CI)	p-value	HR (95%CI)	p-value	HR (95%CI)	p-value
Digital Scoring	3.14 (0.92–10.66)	0.06	4.24 (1.30-13.82)	0.01	0.40 (0.10–1.58)	0.17	0.48 (0.14–1.6)	0.21
Manual Visual Scoring Index	0.84 (0.28–2.52)	0.76	0.85 (0.31–2.36)	0.76	0.68 (0.14–3.24)	0.62	1.03 (0.30–3.48)	0.96
Manual Visual Scoring Intensity	1.44 (0.48–4.33)	0.50	1.523 (0.54–4.22)	0.41	0.57 (0.07–4.59)	0.59	1.43 (0.38–5.30)	0.58
Manual Visual Scoring Area	1.37 (0.43–4.35)	0.58	1.08 (0.36–3.23)	0.88	0.68 (0.14–3.24)	0.62	1.03 (0.30–3.48)	0.96

Finland (n = 147)	Immune hot cases (n = 89)				Immune cold cases (n = 58)			
	Disease specific survival		Overall survival		Disease specific survival		Overall survival	
	HR (95%CI)	p-value	HR (95%CI)	p-value	HR (95%CI)	p-value	HR (95%CI)	p-value
Digital Scoring	1.30 (0.60–2.83)	0.50	1.12 (0.60–2.09)	0.71	1.34 (0.57–3.19)	0.49	1.06 (0.55–2.06)	0.86
Manual Visual Scoring Index	2.50 (1.07–6.06)	0.03	1.68 (0.89–3.19)	0.10	0.74 (0.31–1.76)	0.50	0.98 (0.51–1.92)	0.97
Manual Visual Scoring Intensity	0.61 (0.28–1.33)	0.21	0.58 (0.31–1.07)	0.08	1.08 (0.43–2.56)	0.89	1.13 (0.50–2.25)	0.72
Manual Visual Scoring Area	3.30 (1.14–9.57)	0.02	1.85 (0.90–3.79)	0.08	0.79 (0.32–1.99)	0.62	0.93 (0.43–2.03)	0.86

## Discussion

This multicenter international study sought to validate the prognostic value of B7-H3 in OTSCC, as this immune checkpoint was reported as a prognostic marker twice in head and neck and OSCC [8, 17]. In our OTSCC patient cohort, high B7-H3 expression was associated with poorer prognosis in some national subgroups but only for those whose tumors were highly infiltrated by lymphocytes (immune hot); depending on the scoring method, but it failed to work in the full cohort. A significant association was only found in the univariate but not the multivariate analysis.

Our results highlight a common and serious problem in prognostic marker studies, which can be called the “replication crisis” [22]. Recent systematic reviews of prognostic markers for oral cancer have suggested hundreds of molecules as putative prognostic markers [14, 15, 23, 24]. However, none of them have been adopted into clinical use, and patient management is still mainly based on the clinical TNM staging due to missing or failed validation [15].

In the two previous studies on B7-H3 [8, 17], both patient cohorts were from Asia (Taiwan and Wuhan). National subgroups tend to have exposure differences to risk factors for OSCC, such as heavy tobacco, alcohol, and betel nut use. This explains why results from OSCC cohorts from one part of the world are not necessarily applicable to others. In addition, the samples analyzed in the two previous articles were obtained from the whole oral cavity and the head and neck area while our samples were only from the tongue [8, 17]. In contrast to OTSCC, HPV infection is recognized as an important risk factor for oropharyngeal cancers, underlining the need to distinguish these cancers [25]. Differences in ethnicity and tumor location could be the reason why we failed to validate B7-H3 in our patient cohort. In this study, we collected samples from three different nations (Brazil, Finland, and Norway) representing OTSCC patients of different ethnicities. This resulted in a large sample size combating statistical bias.

To analyze the quality and repeatability of our scoring, we measured inter-rater reliability for manual visual scoring with a κ coefficient. The highest scores and almost perfect agreement were achieved by senior trainees. Junior trainees had the lowest score with moderate agreement. Thus, extra care should be taken when selecting those who score the stained slides and we recommend that the slides evaluation is done by pathologists who have enough expertise in this field.

The field of pathology is rapidly moving towards automated digital scoring and the use of artificial intelligence [26]. In addition to manual visual scoring, we scored the slides using the free automated software QuPath. Use of automated software not only reduces the time for scoring but also increases the reliability of the results and reduces the risk of bias [26]. One of the major challenges of applying this software as a scoring tool is the difference in settings between laboratories and investigators. Thus, we highly recommend that authors publish all adjustable settings to allow others to replicate and validate the work. Even though the digital and manual visual scoring went hand-by hand in the majority of cases, still in some cases they gave different results which call for a better digital and manual visual scoring protocols.

Another serious problem in the field of prognostic marker studies and validation is related to variation of antibody specificity. Theoretically, all antibodies should give similar results if the antibody passes the manufacturer’s quality control. Unfortunately, in practice there are large variations, not just between different antibodies from different manufacturers, but also between different lots from the same manufacturer. For this reason, we tested two antibodies from two different companies, which, fortunately, gave similar staining patterns.

Immune checkpoints, including B7-H3, are group of molecules with effects on immune cells. Recent advancements in cancer immune therapeutics have resulted in tumors being categorized into hot and cold.^6^ Therefore, in this study we investigated lymphocyte infiltration in the invasive area of the tumor tissue. While our survival results were insignificant in the entire cohort, we observed significant results in hot tumors in the univariate analysis. Our findings further indicate that without the affected cells (lymphocytes) in the tumor, the prognostic power of B7-H3 (and likely also other immune checkpoint inhibitors) may be lost. Thus, we stress the necessity of investigating the immune activity of tumors when assessing the prognostic value of B7-H3 expression.

As a conclusion, in this multicenter international study, evaluation of B7-H3 expression revealed prognostic potential for patients with tumors that were highly infiltrated with lymphocytes. However, B7-H3 did not have prognostic value in the whole OTSCC cohort. This study highlighted an important issue in the field of prognostic markers, which is the “replication crisis”. For prognostic studies on immune checkpoints, we encourage researchers to analyze the immune activity of the tumor samples. We also encourage researchers to publish the scoring protocols (either by manual visual or digital scoring) in detail with all adjustable parameters to allow careful replication and validation of the work. Only immunohistochemical markers with prognostic power validated in several research groups and from cohorts of different countries may have the potential to become a useful tool for universal clinical pathology.

## Electronic supplementary material

Below is the link to the electronic supplementary material.

Electronic supplementary material 1 (PDF 5428 kb)
