# Supplementary material for: Evaluation Challenges in the Validation of B7-H3 as Oral Tongue Cancer Prognosticator
Source: Head Neck Pathol. 2020 Sep 21;15(2):469–78. doi: 10.1007/s12105-020-01222-3 (PMC8134649; doi:10.1007/s12105-020-01222-3)
Supplement: Supplementary file 1 — Electronic supplementary material 1 (PDF 5428 kb) [file 12105_2020_1222_MOESM1_ESM.pdf]

Evaluation Challenges in the Validation of B7-H3 as Oral Cancer Prognosticator

Journal: Head and Neck Pathology

Meri Sieviläinen[1,2], Anna Maria Wirsing[3], Aini Hyytiäinen[1,2]*, Rabeia Almahmoudi[1,2]*, Priscila Rodrigues[4,5], Inger-Heidi Bjerkli[3,6], Pirjo Åström[4,5], Sanna Toppila-Salmi[7], Timo Paavonen[8], Ricardo D. Coletta[9], Elin Hadler-Olsen[3,10], Tuula Salo[1,2,4,5,11], Ahmed Al-Samadi[1,2#]

[1] Department of Oral and Maxillofacial Diseases, Clinicum, University of Helsinki, Helsinki, Finland.

[2] Translational Immunology Program, Faculty of Medicine, University of Helsinki, Finland.

[3] Department of Medical Biology, Faculty of Health Sciences, UiT The Arctic University of Norway, Tromsø, Norway.

[4] Cancer Research and Translational Medicine Research Unit, University of Oulu, Oulu, Finland.

[5] Medical Research Center Oulu, Oulu University Hospital, University of Oulu, Oulu, Finland.

[6] Department of Otorhinolaryngology, University Hospital of North Norway, Tromsø, Norway

[7] Skin and Allergy Hospital, Helsinki University Hospital and University of Helsinki, Helsinki, Finland

[8] Department of Pathology, Faculty of Medicine and Health Technology and Fimlab laboratories, Tampere University and Tampere University Hospital, Tampere, Finland.

[9] Department of Oral Diagnosis, Piracicaba Dental School, University of Campinas, Piracicaba, São Paulo, Brazil

[10] The Public Dental Health Competence Center of Northern Norway, Norway.

[11] University of Helsinki Central hospital, Helsinki, Finland.

*Designates an equal contribution to this work

#Corresponding author: Ahmed Al-Samadi, Department of Oral and Maxillofacial Diseases, Clinicum, Biomedicum Helsinki 1, C223b P.O. Box 63 (Haartmaninkatu 8), 00014 University of Helsinki, Helsinki, Finland; E-mail: ahmed.al-samadi@helsinki.fi ; Tel: +358458947224.

SUPPORTING INFORMATION

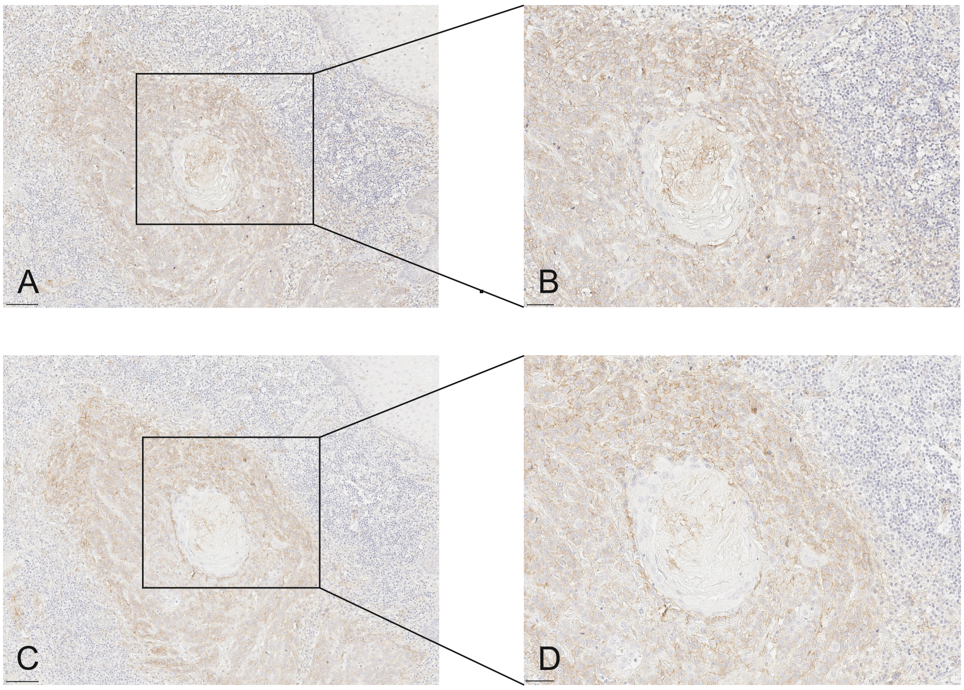

Online resource 1. Two antibodies used in this yielded a similar staining pattern. A-B: goat anti-human B7-H3, C-D: rabbit anti-human B7-H3. Scale bar A and C, 100 µm; B and D, 50 µm.

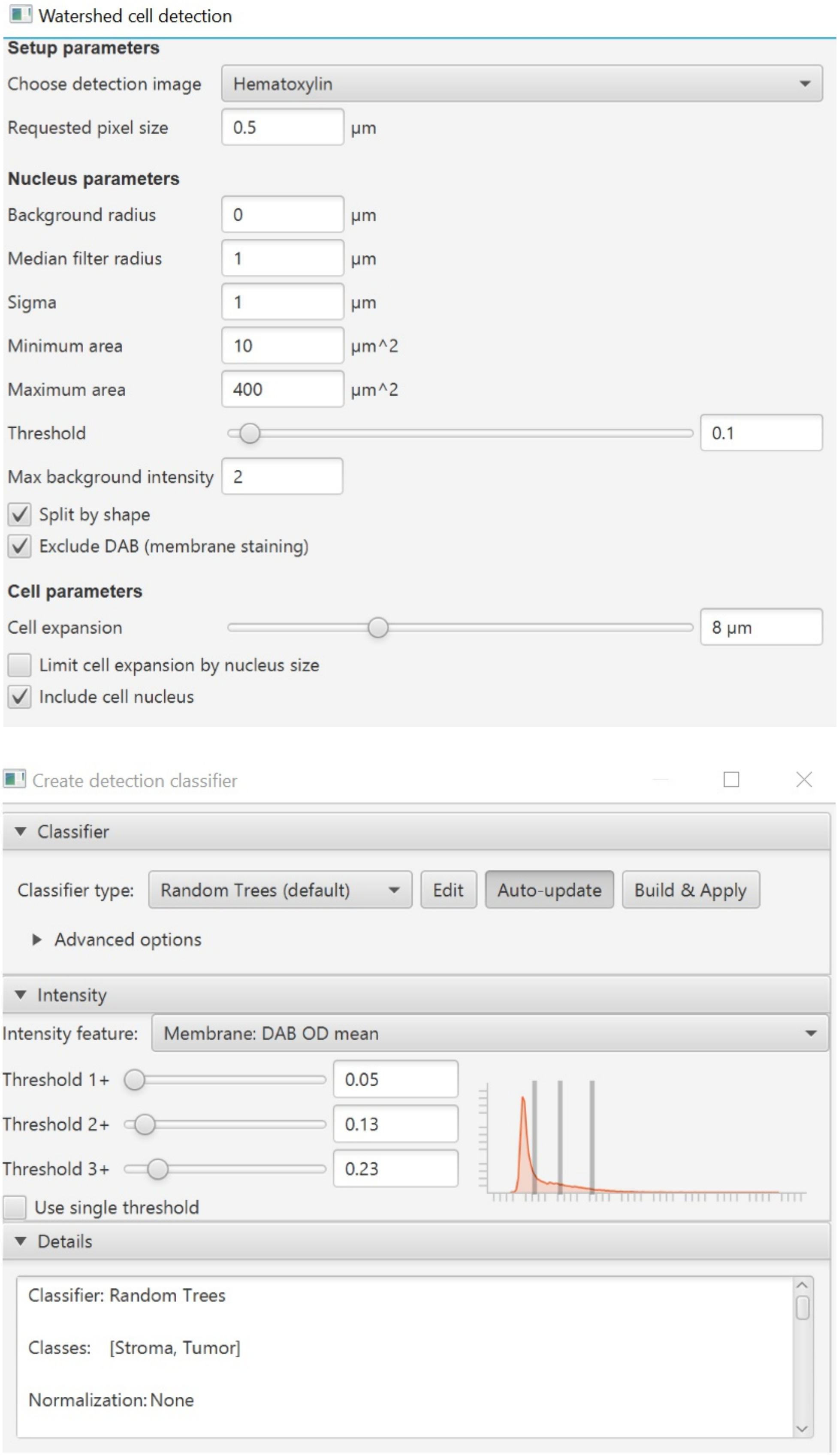

Online resource 2. Scoring parameters for A. cell and membrane detection (threshold 0.1), and for B. membranous DAB classifier (threshold mild staining 0.05, moderate staining 0.13, and strong staining 0.23).

Online Resource 3.groovy

```
setImageType('BRIGHTFIELD_H_DAB');
setColorDeconvolutionStains('{"Name" : "20180228_analysis", "Stain 1" :
"Hematoxylin", "Values 1" : "0.59942 0.56599 0.56599 ", "Stain 2" : "DAB",
"Values 2" : "0.27679 0.48359 0.83038 ", "Background" : " 242 241 241 "}');
selectAnnotations();
runPlugin('qupath.imagej.detect.nuclei.WatershedCellMembraneDetection',
'{"detectionImageBrightfield": "Hematoxylin",  "requestedPixelSizeMicrons": 0.5,
 "backgroundRadiusMicrons": 0.0,  "medianRadiusMicrons": 1.0,  "sigmaMicrons":
1.0,  "minAreaMicrons": 10.0,  "maxAreaMicrons": 400.0,  "threshold": 0.1,
"maxBackground": 2.0,  "watershedPostProcess": true,  "excludeDAB": true,
"cellExpansionMicrons": 8.0,  "limitExpansionByNucleusSize": false,
"includeNuclei": true,  "smoothBoundaries": false,  "makeMeasurements": true}');
runClassifier('C:\\Users\\meris\\Desktop\\b7-h3\\Oulu cases - Priscillawith -
Safecopy\\classifiers\\classifier13319_2.qpclassifier');
```

Online resource 3. Script for Qupath to analyse FFPE slides.

Online Resource 4.groovy

```
setImageType('BRIGHTFIELD_H_DAB');
setColorDeconvolutionStains('{"Name" : "20180228_analysis", "Stain 1" : 
"Hematoxylin", "Values 1" : "0.59942 0.56599 0.56599 ", "Stain 2" : "DAB", 
"Values 2" : "0.27679 0.48359 0.83038 ", "Background" : " 242 241 241 "}');
selectTMACores();
runPlugin('qupath.imagej.detect.nuclei.WatershedCellMembraneDetection', 
'{"detectionImageBrightfield": "Hematoxylin",  "requestedPixelSizeMicrons": 0.5,
 "backgroundRadiusMicrons": 0.0,  "medianRadiusMicrons": 0.0,  "sigmaMicrons": 
1.0,  "minAreaMicrons": 10.0,  "maxAreaMicrons": 1000.0,  "threshold": 0.1,  
"maxBackground": 2.0,  "watershedPostProcess": true,  "excludeDAB": true,  
"cellExpansionMicrons": 8.0,  "limitExpansionByNucleusSize": false,  
"includeNuclei": true,  "smoothBoundaries": false,  "makeMeasurements": true}');
selectTMACores();
runClassifier('C:\\Users\\meris\\Desktop\\b7-h3\\Oulu cases - Priscillawith - 
Safecopy\\classifiers\\classifier13319_2.qpclassifier');
```

Online resource 4. Script for Qupath to analyse TMA slides.
